# Disease-causing gene-flanking genomic rearrangements in HNPCC patients

**DOI:** 10.1186/1897-4287-9-S1-P28

**Published:** 2011-03-10

**Authors:** Monika Morak, Trisari Massdorf, Melanie Locher, Elke Holinski-Feder

**Affiliations:** 1 University Hospital of the Ludwig-Maximilians-University, Campus Innenstadt, Munich, 80336, Germany; 2 MGZ - Center of Medical Genetics, Munich, 80335, Germany

## Background

The molecular diagnosis of hereditary non-polyposis colorectal cancer (HNPCC) or Lynch-Syndrome is the detection of a pathogenic germline mutation in one of the DNA mismatch repair (MMR) genes. However, in ~10-20% of cases suspected of Lynch-syndrome no disease-causing mechanism can be detected. Genomic rearrangements such as gene-flanking deletions, inversions, duplications, or translocations might affect MMR genes - but are difficult to detect. We report here two different disease-causing rearrangement mechanisms in HNPCC patients flanking the gene in question.

## Material and methods

37 patients with colorectal tumours lacking MMR protein staining were included if gene sequencing and deletion screening did not detect MMR germline mutations. The genomic situation was analyzed by oligo array, MLPA kits (P003, P248, P072) and abnormalities were investigated by Long-Range PCRs and sequencing. Additionally, cDNA analyses were performed.

## Results

In one patients with loss of *MLH1* oligo array analyses detected a deletion in *LRRFIP2* exon 20-15, a gene located downstream of *MLH1* with antisense-orientation. The deletion was verified by MLPA in *LRRFIP2* exon 26, *MLH1* exon 1-19 and it`s termination codon were unaffected. The deletion starting after *MLH1* affecting *LRRFIP2* could *per se* not explain the pathogenicity on the *MLH1* gene. In cDNA analyses the coding SNP c.655A>G in *MLH1* exon 8 showed biallelic expression amplifying exons 3-9, 6-9, 3-11, 7/8-14, but monoallelic c.655G in fragments from exon 1-19, 7/8-16, 7/8-18. Suspecting a paracentric inversion we identified two fusion transcripts: *MLH1* exon 1-15 and *LRRFIP2* exon 29 in frame, and *LRRFIP2* exon 1-3 fused with *MLH1* exon 16-19 in frame. The inversion breakpoints in *MLH1* intron 15 and *LRRFIP2* intron 3 with deletion of exons 4-28 were verified in genomic DNA.

A duplication of the complete *MLH1* promoter region and exon 1-19 was found in a patient, his unaffected sister and affected mother. Duplication carriers all showed *MLH1* promoter hypermethylation of 8-18% to 14-25% in DNA from blood, hair follice, colonic and buccal mucosa. We hypothesize that mosaic methylation is a consequence of transcriptional silencing. As cDNA analyses were not informative so far, quantification of *MLH1* expression could not be investigated and no fusion transcripts detected.

## Conclusion

We report disease-causing rearrangements in HNPCC patients: one appeared as a deletion of the *LRRFIP2* gene downstream of *MLH1* but revealed as a paracentric inversion between the two genes creating two new stable fusion transcripts, the other one is a silencing mechanism accompanied with promoter methylation caused by a duplication of the complete *MLH1* gene and flanking region leaving the *MLH1* gene and promotor *per se* intact. In the remaining unsolved HNPCC-suspected patients we expect further mechanisms in the genome to decommission the respective gene which might be detectable by new technologies.

